# The Nursing Supplement

**Published:** 1888-06-30

**Authors:** 


### The Nursing Mirror.

All Contributions for this Supplement should be addressed "The Nursing Mirror," care of The Editor, 38, Old Jewry, E.C.

### En Passant.

WOMEN AS APOTHECARIES.—The Russian Government has granted permission to women to devote themselves to the pharmaceutical profession, provided they pass the same examinations as are established for male candidates. Apothecaries, however, who receive female pupils will not be allowed to take male students also.

THE WOMEN'S LEAGUE.—A similar Congress to that lately held at Washington, under Mrs. Fawcett, is to be shortly held at Paris. For eight days all subjects connected with women and work are to be discussed by hundreds of delegates from all parts of the world. One paper states that there will be 20,000 females, including many lady doctors, but not a single nurse! Why should our profession be so despised by our socialistic sisters?

NURSES IN MANY LANDS.—Princess Christian has consented to be president of the Cyprus Society for the advancement of hospital and educational work in that island. It is proposed to train native nurses there, as Lady Dufferin intends to do in India. We have some Japanese probationers at University College, and in New York they are training some Persian ladies. It is evident that there will soon be no nationality left which cannot boast of that excessively English product, a trained hospital nurse—cap, apron, scissors, forceps, and all.

THOROUGH TRAINING.—As an outgrowth of Prussia's last two wars, when nurses were few and inefficient, the "Albert Verein" Training School was established in Dresden, under the patronage of the Queen of Saxony. The nurses are sent out to private families for three or four marks a day; they also nurse hospitals by contract, and attend the poor gratuitously. The nurses are trained for two years at an asylum and convalescent house in Dresden; they then spend a third year in Leipzig, attending lectures and practical demonstrations. An American book, describing the school, says: "The course is extremely thorough, and if after three years the nurses are invalided in consequence of their duties, they are pensioned." This sounds as though the training was not only thorough but finishing.

ONE OF THE OLDEN TIME.—A correspondent has kindly sent us the following delightful story:—Dr. Crompton, who is, or I am afraid I must say was, one of the oldest doctors in Birmingham, is responsible for the following anecdote. Those who had the pleasure of his acquaintance will remember that he always had a tale apropos of everything. I will give it as nearly as possible in his own words, his own inimitable manner, of course, I cannot give. "When I was a young man I was for a time one of the resident medical officers of the General Hospital. This was long before highly-trained, educated nurses like yourself were thought of; but although not so refined, many of them were good, well-meaning women enough, and did their work fairly well. At any rate, one could depend upon them for one thing, viz., they would carry out the doctor's orders, and would not think that they knew as much or more than the doctors themselves. (Don't disturb yourself nurse, you know I did not mean that for you!) *Revenons à nos moutons.* One day we had received a very bad case of delirium tremens. I was anxious about him, as I knew his chance of recovery was a very slender one, so, before going to bed, I went to see how he was. On walking quietly along the corridor in my slippers I heard a mysterious noise, and what do you think I was just in time to see and hear? Why, the nurse was repeating the Lord's Prayer, and making the man say it after her, a sentence at a time. She had her slipper in her right hand, and between every petition she brought it down on that portion of his body which appears to be considered by mothers and nurses to be specially made and provided by nature for the corporal punishment of infants. When she had finished she thus addressed him. 'There my man, yer can die now as soon as yer like, but I wasn't agoin' to let yer die cussin' the Lord like that!' 'Whatever is the meaning of this, nurse?' I exclaimed. She replied: 'Why his language is that horful it fair makes yer blood curdle, which I thought it would give him a bit of a charnce if he said a prayer before he died.' You see the old woman meant well, although she had a queer way of showing it. Now I don't suppose you would have worried yourself about the man's spiritual condition like that, eh?" I replied, "Well, no, perhaps not; at any rate not to that extent, I think."

THE SHEFFIELD STRIKE.—Miss Corvan, the late superintendent of the Nursing Institute at Sheffield, has secured a comfortable house in that town to serve as a nursing home, and hopes to open it on July 18th. All the nurses who can leave the old institute go with her, and as public feeling is in her favour, there is small doubt that Miss Corvan will be able to efficiently carry on her former good work without being hampered by an unintelligent committee. Perhaps it is not generally known that this committee turned Miss Corvan out of the institution at twenty-four hours' notice, after having accused her of conspiracy. This occurred on June 20th, though Miss Corvan's time is not up till the middle of July. There is certainly something very unaccountable in the proceedings of the committee, and we can only hope that the new lady superintendent may not meet with similar discourtesy at their hands. It will be interesting to notice whether the new home, or the old institute will prove the fittest to survive in the struggle which must inevitaby exist between them.

THE ART OF RESTING.—An evening paper contained an article a little while ago, in which were many remarks applicable to our nurses, who so constantly overwork themselves. It said: "We do not call a man a painter because he daubs colour upon canvas, and women who only exercise their minds upon dress and novels do not know what the delight of doing nothing means. Happily there are scores of women nowadays who use life after another fashion, but even such women, admirable and excellent though they be, never appreciate the delicious art with the relish felt by men. Their sense of responsibility is so great that they seem to dread relaxing the tension of life even for an hour. And yet, if this earth has evils to be fought against and conquered, it has also flowers for scent and birds for song, and many a spot of beauty where the best thing either man or woman can do is to 'rest and be thankful." Dwelling so much, as nurses must, amongst scenes of suffering and pain, it is very necessary for them to seek at times the consolations of inanimate nature, and, in contemplating the beauty of sea, sky, and land, forget for awhile the woes of humanity. We shall never judge this world aright if we always live in one corner of it, and persistently look on life from the same point of view. In the eagerness of learning the profession of nursing, the art of resting must not be forgotten."

### Original Articles.

### NURSING IN NEW YORK.

It is always rather a fearful ordeal for a candidate for nursing to face the matron of the chosen hospital; but the most timid might feel at ease with the lady superintendent of the Bellevue Hospital, New York. A gentle old lady, with gray hair parted in the middle, and uncovered by any cap, and with quiet, faded blue eyes that remind one of pressed speedwells—just that trace of delicate beauty in colouring which suggests what once has been. The hesitating questions of the would-be probationer are answered clearly and kindly, and she leaves with a full knowledge of what she may expect to see and do, should she pursue her fancy for nursing. Bellevue is the large free hospital of New York City, and takes in all cases except infectious fevers. All medical students, after their first years of study, strive to pass a brilliant examination, in order to get admitted to Bellevue: because of its greater opportunities, only candidates of the highest standing are accepted. Of course the nurses have equal powers of seeing and learning with the doctors, so Bellevue is admired as a training school. As to the class of women taken as nurses, out of the 64 accepted this year 29 had been teachers, one had been a dressmaker, one a type-writer, and the others had never before had regular occupation. Servants are never admitted, nor women who have ever served as servants, except in the case of a few Western women of superior education. Such a Scotch servant as Mairi in "A Princess of Thule," for instance, might have the rule relaxed in her favour. No married women are eligible unless they are widows. Those who have had the management of households are welcome at Bellevue, and can nearly always procure appointments as superintendents. Women of refinement are especially desired, and strange to say, those who look most delicate often prove the toughest and best nurses. There are not many ladies of independent means in Bellevue; the menial duties and strict *régime* discourage them. Yet American women are not educated up the standard of comfort prevalent in England: the very climate makes their lives uncomfortable. They are frozen to death in winter, and frizzled in summer—gasping for air like fishes out of water. In that position science can do little for them. Life is always at high pressure, too, in New York—everyone is in a hurry, and the head swims to see the people whirling around so. The doctors in America are very kind and considerate to all women, and do their best to lighten the trying lives of the nurses.

After the trial month the nurse-probationer has to pass a simple examination in the three R's. Those who prove satisfactory, agree to remain two years at the training school, and to obey all its rules. The pay for the first year is seven dollars a-month, for the second year twelve dollars a-month, which sum is given merely to cover the expenses of dress, books, &c., and is in nowise intended as wages. A certificated nurse can generally earn about £4 a-week. Day nurses in Bellevue are on duty from eight a.m. to eight p.m., with an hour for dinner, and time off when possible. They always have one afternoon free in the week, and a vacation of two weeks in the summer. The institution is unsectarian, but there are daily prayers, which the nurses attend, and they are all expected to go to service at their chosen place of worship once every Sunday. The course of training is admirable, and includes (1) The dressing of blisters, burns, sores, and wounds; the application of fomentations, poultices, cups, and leeches; (2) The administration of enemas, and use of cathether; (3) the management of appliances for uterine complaints; (4) the best method of friction to the body and extremities; (5) the management of helpless patients—making beds, moving, changing, giving bath in bed, and preventing and dressing bed-sores; (6) bandaging, making bandages and rollers, lining of splints; (7) the preparing, cooking, and serving of delicacies for the sick. The nurses are also given instruction in the best practical methods of supplying fresh air, warming and ventilating sick-rooms in a proper manner; and are taught to take care of rooms and wards; to keep all utensils perfectly clean and disinfected; to make accurate observations and reports to the physician of the state of the secretions, expectoration, pulse, skin, appetite, temperature of the body, intelligence—as delirium or stupor—breathing, sleep, condition of wounds, eruptions, formation of matter, effect of diet, or of stimulants, or of medicines; and to learn the management of convalescents. The teaching is given by visiting and resident physicians and surgeons at the bedside of the patients, and by the superintendent, assistant superintendent, and head nurses. Lectures, recitations, and demonstrations take place from time to time, and examinations at stated periods.

There is always an opening address to the graduating class, delivered by the chief physician; and the regular lectures last year included four courses, on "Obstetrics," "Anatomy," "Diseases," and "Physiology."

When the full term of two years is ended, the nurses thus trained can choose their own field of labour, whether in hospitals, in private families, or in district nursing among the poor. On leaving the school, after passing the final examination, each receives a diploma signed by the examining board, and by a committee of the board of managers.

But the school does not lose sight of its graduates, as we foolishly do in England; a complete list is kept, and there one can read the present fate of all the nurses who have ever trained at Bellevue for the last thirteen years. Thus we learn that Miss Martin graduated in 1882, and is now superintendent of St. Paul's Home, Rome; that Miss Brown graduated 1878, took M.D. degree 1885, married Dr. Dewey, and now lives at Kanhatree, Ill.; that Miss Allen graduated in 1881, and is now matron of a lying-in hospital in London; that Miss Vernois graduated in 1878, and is now a district nurse in Holland. What a pity all training schools do not keep such a register!

A prominent physician connected with Bellevue says he considers nursing the most advantageous profession for women. A capable woman, in ten years, or fewer, is independent. Her presents outside her pay are often very handsome. As a general rule, she marries well within a couple of years, and leaves a vacancy for the next capable woman. English nurses are much in request in America, their training being held in the highest estimation. Before entering on their career in a new country, however, it is best for them to have an introduction to some prominent physician, or be in correspondence with one, in order to avoid disappointment and tedious waiting. They will find the patients in American hospitals rather different from our English patients. It must be remembered that the word "humility" has been erased from the American dictionary, and that gratitude there may be, but humble thanks never. In private work, nurses always receive the greatest consideration and munificence; but everything is much more expensive in New York than in London, and the climate and living are very trying to strangers. There are always two sides to a question.

---

One who is hesitating whether or not to undertake the work of friendly visiting as a Christian duty, may be helped by remembering that the most practical of the Apostles has said: "Pure religion and undefiled before God and the Father is this, to *visit* the fatherless and widow in their affliction, and to keep himself unspotted from the world." It is not enough to give alms to the poor; religion requires that we should come into personal contact with them, and render them, according to our opportunities and our ability, personal service.

### Everybody's Opinion.

[*Correspondence on all subjects is welcome, but correspondents would oblige by writing on one side of the paper only.*]

### NURSES AND PATIENTS.

Our correspondent, "James Macfarlane," has brought a shower of indignation down on his devoted head, *via* The Hospital office, by his unflattering estimate of nurses. With regard to his declaration that he would not choose a wife from among those he knows, there seems to be but one reply, coming unanimously from every quarter of the country —that contained in the old song, "Nobody asked you, sir, she said." But not only nurses, but patients, cherish a different opinion from his, as the following letters show :

### A Retort from Manchester.

You have all sorts writing to you, as your correspondent, James Macfarlane, says in last week's Nursing Supplement; but I think if he had left out those ungracious remarks about nurses it would have been more to his credit. He asks what hospitals would be without patients? Let him ask himself what they would be without nurses, and what the patients would do without them? I have been a patient twice in a hospital, and I can say that nurses have a great deal to do. They cannot please everyone, and it is always the most ignorant ones who are the most difficult to please. They are never satisfied ; they think a nurse must be at their beck and call, just as if there were one for each bed, instead of one for ten, or perhaps twenty beds. Your correspondent says that when he hears a man groaning it is worse to him than his own pain; but the nurse does not seem to care. I am sure that is untrue, and I think he is without feeling himself when he speaks in such a manner. They may not show how much they mind, but they feel it, nevertheless. But the bravest of them show it the least, and the kindest of them try to keep our hearts up, even though we may be at death's door. He also says that he would not choose a wife out of any of those that have waited on him. Perhaps, if the truth were known, none of them would choose him for a husband.

E. G.

### An Answer from Devon.

I have just taken the Nursing Supplement to read, and find in it that a patient speaks of the nurses in hospitals, as he terms it, chucking aside the care of patients, and not taking enough notice of them. What does he want? Would he like a nurse to be sitting by his bedside night and day, and to be always sympathising and petting him like a child? Nurses do see sad sights, and it is their duty to see all the operations. But for all that they must have got the same feeling as anyone else. I myself have heard men groaning, but I cannot see how it can make any difference to a patient's pain to hear others groaning. But as for the nurses taking no heed of the patients, I can speak from my own experience. I have been in hospital close on five months, and have had all the attendance that anyone could wish for. If it had been my own mother that had been nursing me I could not have been treated better. I, myself, consider James Macfarlane's remarks on choosing a wife most ridiculous. Does he think nurses in general take up that vocation on the chance of getting married, and care to be, as he said, picked up as a wife?

E. A.

### The Nurse Speaks.

If nurses were what James Macfarlane would have them, our lives would not be very pleasant, neither would we ever be able to overtake our trying tasks. The writer says he won't take a wife from our ranks. We beg to state that nurses are not husband-hunters. I would have the writer to know that women as soft as asparagus will not make brave nurses. A good nurse can be gentle and kind, and at the same time she must be firm; and if nurses don't carry their sympathy on their dress sleeve at every sad sight they see, that does not go to say that we are void of feeling. The writer must learn that a cheerful and bright nurse is a great help to patients, whereas a nurse that would always be sorrowing with the patients, having a look on her face as if she went to meet trouble half way, would be a great drawback.

Nurse P.

### A Probationer's View.

As you so kindly provide accommodation in your delightful paper for "Everybody's Opinion," will you allow a probationer to express hers on the feelings which exist between nurses and their patients, as referred to by a correspondent in the number of June 16th inst. He complains of a want of sympathy on the part of nurses, but I believe they have a great deal more of this in feeling than in expression. Some nurses have such strange manners that I do not wonder that their best intentions are sometimes misunderstood. Many will probably think it at all events very "cheeky" for a pro. to express her opinion on such subjects; but if probationers do not always excel in practical knowledge, they may still, by observation, acquire a good deal of insight into the characters of those around them : and who better than a probationer has the chance when the "staff nurse "is off duty, or not near at the time, of hearing the little remarks about "our nurse?" Of course I am not excluding probationers from my ideas—we have equally to share the goodwill and censure of our patients; but just as a staff nurse is answerable for the "tone" of her ward, so is she in a great degree for the "style" of her probationer. I have been working in a large London hospital, and under many different nurses. May I be allowed to classify them as they have appeared to my mind? 1st. There is the nurse who makes everyone feel happy and good tempered—just because she is so herself. There is always a bright word for every bed she stops at; nothing that she has to do gives her trouble, and somehow her patients never seem to give so much trouble as others do. She has the knack of making her sympathy felt, even if she does not express it, and they love her for it, too, and are not afraid to say so. Then there is that extremely conscientious never-tiring nurse, who by her unceasing attentions wins the deepest respect of all her patients, but at the same time has so many things to think of, or has such an irritable mania for putting things to rights, that though often full of sympathetic feeling for her patients, she thinks she has no time to bestow it on anyone. Then, alas! I fear there is sometimes the "nagging," ill-tempered nurse, who may be ever so anxious to do her duty, but who makes a worry of everything beyond the regular routine of ward work. Oh, how I pity her poor patients whose every want supplied is preceded by a cross word of remonstrance, and often followed by a scolding for giving so much trouble! There may, indeed, be but few of these—'tis well if so—but how unconsciously and unintentionally do many of us incline to that way of showing our patients when they give trouble. And after all, what is a nurse's duty but the undertaking of works of trouble—not performed as such, but transformed, by the very object of her profession, into works of love? Oh, how much happier, whilst even suffering, would be the faces in our wards, how much more keenly our sympathy be felt by those for whom we labour, could we learn in all things, however trivial, to do our deeds as if we loved to do them, and not because we must.

A Probationer.

### PRESENCE OF MIND *VERSUS* PANIC.—A REPLY.

Aidyl sincerely hopes that all readers of her "Night's Adventure" have not judged her as harshly as "A Matron" appears to have done. The event described occurred shortly after the execution of the notorious Charles Peace, whose fearful crimes were at that time a household topic. Aidyl had just completed her hospital training, and was prepared to meet the emergencies of disease and death with a calm front; but the unthought-of terror of a midnight burglar took her quite by surprise. Since then her courage as a nurse has stood the test of nine years' nursing without any grave charge being laid at her door. Aidyl is glad to say she has never had another alarm of the same kind to try her nerves.

### Appointments.

[It is requested that successful candidates will send a copy of their applications and testimonials, with date of election, to The Editor, The Lodge, Porchester Square, W.]

Miss Charlotte Prince has been appointed matron of the Blackburn Infirmary, out of forty-eight candidates. Miss Prince was trained at St. Thomas's, and was a sister there for three years. She then spent some time abroad in the service of a sick friend, and since her return to England has taken several holiday duties. Twice she has acted for the matron of the Northern Hospital, Liverpool, and once for the matron of the Borough Hospital, Birkenhead. Miss Prince went to Blackburn in April as temporary matron, and was elected to the permanent appointment on June 4th. After all her wanderings, we hope Miss Prince has found a comfortable home.

Miss Florence Nott Bower has been appointed matron of the Huddersfield Infirmary, which is about to be enlarged by the addition of a new wing, to hold sixty more beds. Miss Bower trained as a lady pupil at Guy's Hospital five years ago, and has since had charge of various wards. For the last year Miss Bower has acted as Sister Dorcas—Dorcas being the obstetrical ward at "Guy's."

Miss H. Lawrence was elected Matron of the Dorset County Hospital on the 14th of this month. Miss Lawrence trained eight years ago at Addenbrookes, Cambridge, and for three years had charge of the male surgical ward there. Afterwards she became sister at the Royal Hants County Hospital, and later, night superintendent at Salisbury. For the last few months Miss Lawrence has been working at the Maidstone Hospital, giving particular care to the operating theatre. Miss Lawrence has many excellent testimonials, and we hope they have secured her a pleasant post.

### Honours and Presentations.

[Information for this Column should be addressed to The Editor, The Lodge, Porchester Square, W.]

The night nurses of the Bristol Royal Infirmary have presented a handsome clock to their late Night Superintendent, Miss Stevenson, whose appointment as Matron of Bradford Children's Hospital we reported last week. During the two years that Miss Stevenson has filled the post of Night Superintendent at Bristol she has particularly endeared herself to the nurses under her charge, and it is with feelings of the deepest regret that they see her depart for a fresh sphere of labour. This will be pleasant reading for the Committee who selected Miss Stevenson from a large number of applicants, and may be regarded as a happy augury of the success that awaits her at Bradford.

### The Nurses' Bookshelf.

### Hints to Cottage Hospital Matrons.*

This is a small pamphlet on nursing, anatomy, dispensing, and housekeeping in cottage hospitals. It contains much useful information, but we think the author can scarcely have considered how slender is the purse of the average nurse, when she puts in the list of useful books Witkowski's "Human Anatomy and Physiology, with Movable Atlases of the Human Body," in ten parts, each part 7*s.* 6*d.* All the anatomy nurses need can be learned from a much cheaper manual than this, as Miss Heanley proves by devoting only two pages of the pamphlet to her subject. Miss Heanley evidently knows the subject she writes about thoroughly, but she still is wanting in the art of putting her knowledge clearly as well as briefly before her readers. This is a point which practice in writing will doubtless improve. This pamphlet has, however, many merits, one of which is its very low price.

### Vacancies.

The following vacancies are announced :—

### Matrons.

Gateshead Children's Hospital ; £35. Worcester Infirmary ; £80. Swindon Victoria Hospital.

### Lady Superintendents.

Shrewsbury Infirmary ; £100. Bristol Children's Hospital. Salop Infirmary.

### Nurse Housekeeper.

Infectious Hospital, Liverpool ; £30.

### Nurses.

Huddersfield Infirmary ; two ; £24 and £18.

City of London Hospital, Victoria Park ; £20.

Bradford Children's Hospital ; £22.

Carlisle Infirmary ; two.

Croydon Union ; £22.

County Institution, Worcester.

Blackburn and East Lancashire Infirmary. £27.

Glasgow Sick Poor and Private Nursing Association. Several.

Maidstone Hospital ; four ; £25 and £16.

Darenth Imbecile Asylum ; £25.

Hoxton House Asylum ; £20.

Southport Nurses' Home.

Chelsea Hospital for Women ; £20.

Kingston Home, Hull.

District Nurses' Home, Leeds.

Royal Albert Hospital Devonport Nursing Institute. £25.

Eccles and Patricroft Hospital. £20.

### Probationers.

Stafford Infirmary ; two ; no salary.

### Notes and Queries.

To Correspondents.—1. Questions may be written on post-cards. 2. Advertisements in disguise are inadmissible. 3. In answering a query please quote the number. 4. If a private answer is desired, a stamped addressed envelope must be forwarded.

(32) Nurse Frances would be glad to know where she could obtain "The Life and Work of Agnes Jones."

(33) Will any reader kindly give information how a Q. C. H.'s midwife can get a post abroad as such, and is it not lucrative? She has also experience of a few months' general nursing.—*Adita, Nurse.*

* "Hints for Matrons and Pupils in Cottage Hospitals," by K. M. Heanley

